# Exploring the Impact of Socially Assistive Robots in Rehabilitation Scenarios

**DOI:** 10.3390/bioengineering12020204

**Published:** 2025-02-19

**Authors:** Arianna Carnevale, Alessandra Raso, Carla Antonacci, Letizia Mancini, Alessandra Corradini, Alice Ceccaroli, Carlo Casciaro, Vincenzo Candela, Alessandro de Sire, Pieter D’Hooghe, Umile Giuseppe Longo

**Affiliations:** 1 Fondazione Policlinico Universitario Campus Bio-Medico, Via Álvaro del Portillo, 200, 00128 Roma, Italy; arianna.carnevale@policlinicocampus.it (A.C.); alessandraraso@hotmail.it (A.R.); carla.antonacci@unicampus.it (C.A.); l.mancini@policlinicocampus.it (L.M.); a.corradini@policlinicocampus.it (A.C.); a.ceccaroli@policlinicocampus.it (A.C.); c.casciaro@policlinicocampus.it (C.C.); v.candela@policlinicocampus.it (V.C.); 2 Laboratory of Measurement and Biomedical Instrumentation, Department of Engineering, Università Campus Bio-Medico di Roma, Via Álvaro del Portillo, 21, 00128 Roma, Italy; 3 Department of Medical and Surgical Sciences, University of Catanzaro “Magna Graecia”, 88100 Catanzaro, Italy; alessandro.desire@unicz.it; 4 Research Center on Musculoskeletal Health, MusculoSkeletalHealth@UMG, University of Catanzaro “Magna Graecia”, 88100 Catanzaro, Italy; 5 Department of Orthopaedic Surgery and Sportsmedicine, Aspetar Hospital, Doha 29222, Qatar; pieter.dhooghe@aspetar.com; 6 Research Unit of Orthopaedic and Trauma Surgery, Department of Medicine and Surgery, Università Campus Bio-Medico di Roma, Via Alvaro del Portillo, 21, 00128 Roma, Italy

**Keywords:** socially assistive robots, rehabilitation, robot-assisted therapy, motivation, physical therapy, patient engagement

## Abstract

Background: Socially Assistive Robots (SARs) represent an innovative approach in rehabilitation technology, significantly enhancing the support and motivation for individuals across diverse rehabilitation settings. Despite their growing utilization, especially in stroke recovery and pediatric rehabilitation, their potential in musculoskeletal and orthopedic rehabilitation remains largely underexplored. Although there is methodological and outcome variability across the included studies, this review aims to critically evaluate and summarize the research on SARs in rehabilitation, providing a thorough overview of the current evidence and practical applications. Methods: A comprehensive search was conducted across multiple databases, resulting in the selection of 20 studies for analysis. The reviewed papers were categorized into three main classes based on the roles of the robots in rehabilitation: Motivation, Imitation, and Feedback Providers. Results: The analysis highlights that SARs significantly improve adherence to rehabilitation programs, enhance motor function, and increase motivation across clinical and home settings. Robots such as NAO, Pepper, and ZORA demonstrated high efficacy, particularly in stroke recovery and pediatric rehabilitation. Conclusions: SARs offer transformative benefits in rehabilitation, providing scalable, personalized solutions through motivational support, guided exercises, and real-time feedback. Their integration into orthopedic rehabilitation could address critical clinical needs, enhancing precision in exercises, adherence to long-term programs, and overall patient outcomes. Future research should prioritize the development and validation of SAR-based interventions for musculoskeletal disorders to unlock their full potential in this domain.

## 1. Introduction

Robotics, as a technological domain, encompasses the design, operation, and application of computer systems, electronics, and software programming to perform tasks involving information processing, signal control, and feedback measurement [1]. This field has led to the development of automated machines capable of replicating or replacing human functions across various industries. In recent years, a significant rise in robotic applications has been observed in rehabilitation and assistive technology [1,2]. The increased utilization can be attributed to the robots’ ability to provide consistent, repeatable movements, offer treatments with controlled intensity, and engage patients in their therapy, ultimately improving their independence and social interaction [2,3]. Additionally, robot-assisted therapy enables rehabilitation teams, including physiotherapists, physicians, and bioengineers, to customize therapy by adjusting parameters like exercise type, assistance level, and kinematics [4,5]. Robots can assist psychologically and physically, especially when an affected limb is no longer functional.

In the past decade, research into social robots capable of autonomous interaction has expanded [6,7,8,9]. Among these, Assistive Robots and Socially Assistive Robots (SARs) have seen significant development. Assistive Robots are primarily designed to provide physical support or assistance to patients during rehabilitation or daily activities [10,11]. These robots typically interact with users through mechanical means, such as offering resistance during exercises, supporting movements, or compensating for physical disabilities (e.g., robotic exoskeletons or end-effectors used in rehabilitation) [12,13,14]. Their primary focus is on enhancing motor recovery and improving physical function [10]. On the other hand, SARs aim to provide social and therapeutic support without direct physical interaction [14]. They often feature personalized interactions, encouragement, and real-time feedback, creating a more engaging and supportive therapeutic environment. SARs have shown great potential in engaging patients in various therapeutic settings, including autism treatment and rehabilitation exercises [12,15,16,17]. Their growing relevance is also driven by the rising elderly population and the increased incidence of conditions such as strokes and neurodegenerative diseases, which call for scalable, effective rehabilitation solutions [18,19,20]. SARs are primarily utilized to enhance motor and cognitive functions, motivate patients, and improve overall quality of life, especially for individuals recovering from strokes, managing chronic diseases, or with musculoskeletal disorders. These robots employ communication techniques like gestures, speech, and eye contact to engage patients, guiding them through the rehabilitation process. They have been developed in multiple forms and designs, serving specialized purposes across different interventions, including rehabilitation, treatment, and diagnostics. Humanoid robots, animal-like robots, toy robots, and robotics kits are just a few examples [17,21]. Research has shown that intensive and repetitive practice of functional tasks is essential for recovery, and SARs provide an effective way to deliver this type of therapy [22]. By keeping patients motivated and offering real-time feedback, SARs improve treatment adherence and reduce the need for constant human supervision [23]. This ability to offer continuous, personalized care makes them invaluable in rehabilitation, not only supporting patients but also relieving caregivers from more routine tasks so they can focus on more complex aspects of care. Given the increasing demand for rehabilitation services, SARs play a pivotal role in lightening the workload for healthcare professionals and caregivers [10]. These robots deliver real-time feedback, monitor progress, and provide personalized therapy in both clinical and home environments, enhancing scalability and effectiveness in rehabilitation programs [22]. Overall, the use of SARs in rehabilitation signifies a promising advancement in healthcare and robotics, presenting innovative methods to engage and support patients while enhancing the scalability and efficiency of rehabilitation.

While other reviews have addressed SARs in fields such as mental health, pain management for children, and care for the elderly [24,25,26], this review primarily focuses on their application in motor rehabilitation, emphasizing their role in supporting patients’ physical recovery and functional improvement. Despite the heterogeneity of the included studies—varying in methodologies, sample sizes, and outcome measures—this review aims to critically evaluate and synthesize research on SARs in rehabilitation settings, providing a comprehensive overview of current findings and practical applications. Moreover, certain rehabilitation domains, such as musculoskeletal and orthopedic conditions, remain underexplored in the existing literature, highlighting important avenues for future investigation.

## 2. Materials and Methods

This systematic review was conducted following the PRISMA guidelines [27]. The protocol of the systematic review was officially registered in the Open Science Framework (OSF) database, with the registration DOI: https://doi.org/10.17605/OSF.IO/6XECG, accessed on 28 January 2025. This paper was focused on qualitatively synthesizing and summarizing the existing literature without employing advanced statistical methods or quantitative analysis [28,29]. The literature search was performed across multiple databases, including PubMed, IEEE Xplore, and Web of Science, to ensure comprehensive coverage of relevant studies published between 2008 and September 2024. The search encompassed a range of conferences, journals, and technical publications. The database search query was designed around two key concepts: the intervention (Socially Assistive Robots, SARs) and the context (rehabilitation). Free-text terms and Boolean operators (AND, OR) were used to construct the search strategy. The following keywords were applied individually and in combination: “Socially Assistive Robots”, “Rehabilitation”, and “Robot-Assisted Therapy. In detail, in each database, the search was executed as follows: (“Socially Assistive Robots” OR “rehabilitation robots” OR “robot-assisted therapy” OR “robotic rehabilitation” OR “robotic therapy” OR “assistive robots” OR “socially assistive technology”) AND (“rehabilitation” OR “physical therapy” OR “occupational therapy” OR “motor rehabilitation” OR “neurorehabilitation”) AND (“robot-assisted therapy” OR “robotic intervention” OR “robotic training” OR “robot-assisted rehabilitation”). This strategy ensured that all potential studies involving SARs and their application in rehabilitation were included, provided they met the following inclusion criteria:

1. The study must exclusively address rehabilitation, including only research that explicitly focuses on rehabilitation practices.
2. The study must incorporate the use of one or more robots in the rehabilitation process for individuals with various conditions, specifically utilizing SARs as a core component of the therapeutic or rehabilitative intervention.
3. The study must incorporate the use of one or more robots in the rehabilitation process for individuals with various conditions, specifically utilizing socially assistive robots (SARs) as a core component of the therapeutic or rehabilitative intervention.

**Figure 1:**
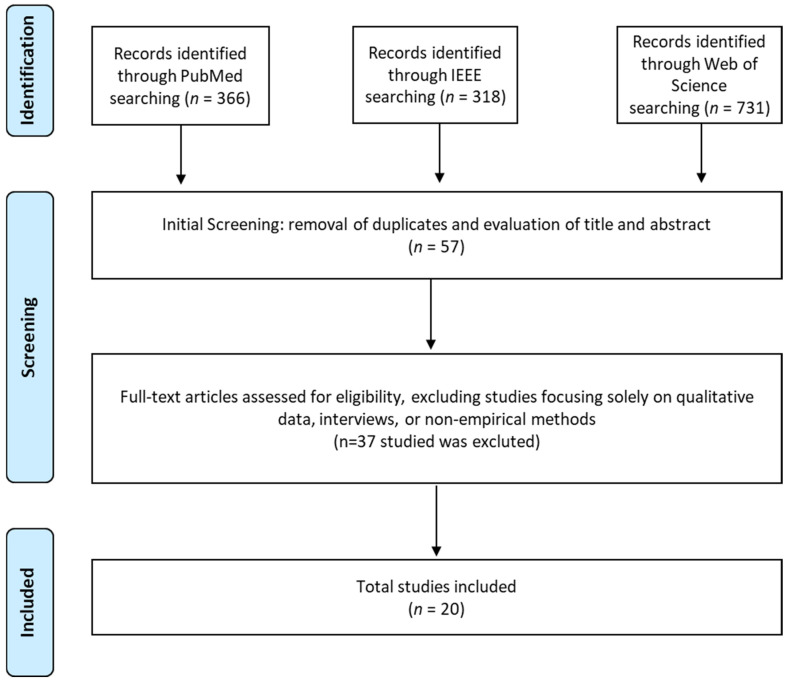
PRISMA flow diagram.

The selection process is summarized in Figure 1. After duplicates were removed, the titles and abstracts of the articles retrieved from the search were independently screened by three reviewers. The same reviewers conducted a detailed assessment of each full-text article considered for inclusion. In cases of disagreement, the final decision on inclusion or exclusion was reached through discussion with three additional reviewers. From the initial screening process, 57 studies were identified as relevant to the review. Following a more detailed examination, 37 articles were excluded due to their focus on qualitative assessments rather than rehabilitation or reliance solely on interviews or non-empirical data.

After this filtering process, 20 studies were deemed eligible and were analyzed in full. Additionally, the reviewed articles were grouped into different categories. These categories highlighted the diverse roles of robots in rehabilitation, showcasing their potential to enhance therapeutic engagement, learning, and outcomes. The three categories were detailed as follows:

1. Studies focusing on motivation, where robots were used to enhance patient engagement during therapy. This category included studies where robots were primarily used to enhance patient motivation and encouragement during rehabilitation activities. Their primary role was to offer emotional and motivational support, fostering user engagement through intelligent interactions designed to improve health and psychological well-being by providing companionship.
2. Studies emphasize imitation, where robots demonstrate exercises for patients to replicate. In this category, robots were employed to demonstrate specific rehabilitation exercises, which patients were required to imitate. The primary objective was to guide patients through movements, ensuring they replicated the robot’s actions as accurately as possible.
3. Studies leveraging feedback, where robots provided real-time corrections and guidance to patients during rehabilitation. This category included studies where robots were utilized to provide corrections and feedback on the movements performed by patients during rehabilitation exercises, monitoring and improving their performance. The robots in these studies played a crucial role in ensuring that patients performed the exercises accurately, offering real-time guidance and adjustments.

## 3. Results

The characteristics of the reviewed studies, including titles, publication years, robots used, patient populations, and study aims, are summarized in Table 1.

A total of 225 patients with different characteristics were identified across the selected articles. The included studies addressed a variety of pathologies, with 40% focusing on neurological disorders, 20% on cardiovascular rehabilitation, 25% on motor or physical disabilities, and 15% on other types of pathologies. Regarding the age distribution of participants, 50% of the studies involved adults aged 18 to 65 years, 30% focused on individuals over 65 years, and 20% included pediatric populations under 18 years. The heterogeneity of the studies was also evident in the rehabilitation outcomes associated with the use of SARs. Specifically, 70% of the studies reported substantial improvements in patient rehabilitation, 20% showed moderate enhancements in recovery, and 10% found no significant or measurable effects.

Additionally, in this review, the studies were grouped into three primary categories based on the roles of SARs in rehabilitation: motivation, imitation, and feedback providers. The analysis of the categorized studies revealed notable heterogeneity in their approaches and objectives. Specifically, eight studies focused on motivation, emphasizing the role of robots in enhancing patient engagement and therapeutic adherence [30,31,32,33,34,35,36,37].

Robots such as NAO and Pepper provided continuous verbal feedback, encouragement, and gamified exercises to motivate patients, thereby reducing the need for constant therapist supervision. For example, in cardiac rehabilitation, studies by Céspedes et al. and Casas et al. reported improved patient adherence and faster recovery due to NAO’s motivational feedback [30,31]. In the study by Casas et al., NAO provided personalized motivational support during physical exercises in cardiac rehabilitation, improving patient adherence to the rehabilitation program [33]. Similarly, Buitrago et al. found that NAO enhanced walking performance in a child with cerebral palsy through regular encouragement and personalized guidance [37]. In elderly rehabilitation, Pérez et al. noted that SARs contributed to better physical and mental activity engagement, improving the overall quality of life [32]. Additionally, Meyer et al. and Irfan et al. highlighted the positive impact of motivational robots on patient confidence and participation during therapy sessions [34,35]. Polak et al. explored the use of Pepper in gamified upper-limb rehabilitation exercises, demonstrating enhanced patient engagement and bilateral hand use [36].

Four studies centered on imitation, where robots demonstrated exercises for patients to replicate, fostering learning and motor skill acquisition [38,39,40,41]. For instance, Carrillo et al. showed how NAO effectively led pediatric rehabilitation sessions for children with cerebral palsy, enabling more autonomous therapy [38]. Similarly, Van den Heuvel et al. demonstrated that ZORA’s imitation exercises improved motor skills and engagement in children with severe disabilities, although the novelty of the robot diminished over time [39]. Butchart et al. and Malik et al. highlighted NAO’s ability to increase motivation and participation in therapy for children with cerebral palsy while receiving positive feedback from parents and therapists [40,41].

Lastly, eight studies were classified as feedback providers, leveraging robotic systems to deliver real-time corrections and guidance, thereby optimizing movement accuracy and rehabilitation outcomes [42,43,44,45,46,47,48,49]. For example, Tapus et al. demonstrated that SARs tailored to patient personality traits enhanced exercise performance during post-stroke rehabilitation [42].

Bryant et al. found that children preferred NAO’s corrective feedback in a virtual reality game over human feedback, which increased motivation and improved movement accuracy [43]. Sobrepera et al. developed Lil’Flo to monitor upper extremity motor rehabilitation, providing effective real-time feedback and a more interactive experience than traditional systems [44]. Polak et al. and Matarić et al. reported significant clinical improvements in stroke rehabilitation, with SARs enhancing measures like the Fugl-Meyer Upper Extremity Assessment (FMA-UE) and the Action Research Arm Test (ARAT) [45,46]. Similarly, in the study by Pulido et al., the NAO robot demonstrated poses for pediatric patients with motor disabilities [48]. The robot provided verbal feedback and corrective actions as needed, focusing on keeping the children engaged in their exercises without the need for continuous human intervention. Additionally, Lee et al. showed that NAO’s real-time corrections achieved accuracy comparable to expert assessments, improving patient confidence and engagement [49].

**Table 1:** Summary of included studies.

Title	Year	Robot	Participants			Aim and Methods	Findings
			#	Diagnosis	Age		
A Socially Assistive Robot for Long-Term Cardiac Rehabilitation in the Real World [30]	2021	NAO	20	Cardiac diseases	43–80 years	Evaluate NAO’s role in motivating long-term cardiac rehabilitation by providing continuous feedback and encouragement	Patients in the robot-assisted group showed improved adherence and faster recovery in heart rate and physical performance
Architecture for a Social Assistive Robot in Cardiac Rehabilitation [31]	2018	NAO	1	Myocardial infarction	55 years	Test NAO’s ability to monitor and correct posture during cardiac rehabilitation, offering support and motivational feedback	Robot successfully monitored and corrected posture; patient reported positive experience, but response time could be improved
Caregiver and Social Assistant Robot for Rehabilitation and Coaching for the Elderly [32]	2015	LEGO Mindstorms NXT	28	Elderly participants	-	Develop a robot to coach elderly people in physical and mental activities to maintain healthy habits	Participants found the robot easy to use and motivating, with high satisfaction reported in questionnaires
Social Assistive Robots: Assessing the Impact of a Training Assistant Robot in Cardiac Rehabilitation [33]	2020	NAO	6	Myocardial infarction or coronary bypass	58 years	Evaluate NAO’s impact on motivation during phase II cardiac rehabilitation by providing continuous feedback and monitoring	Robot increased patient motivation and reduced heart rate at rest compared to the control group
Robotic Companions in Stroke Therapy: A User Study on the Efficacy of Assistive Robotics among 30 Patients in Neurological Rehabilitation [34]	2017	ROSEAS	30	Stroke	Various ages	Assess ROREAS in guiding stroke patients during walking exercises, improving motivation and self-confidence	60% of patients preferred robot-assisted walking exercises; confidence and motivation increased during the intervention
Using a Personalised Socially Assistive Robot for Cardiac Rehabilitation: A Long-Term Case Study [35]	2020	NAO	1	Myocardial infarction	60 years	Evaluate NAO’s personalized feedback in motivating a patient during long-term cardiac rehabilitation	Robot successfully identified a critical health event, improved recovery and adherence over 35 sessions
Novel Gamified System for Post-Stroke Upper-Limb Rehabilitation Using a Social Robot [36]	2022	Pepper	12	Clinicians expert in post-stroke rehabilitation	-	Explore Pepper’s use in gamified rehabilitation for upper limb post-stroke, using functional movement games	Clinicians found the system engaging; suggestions included adding bilateral hand use options
A Framework for User Adaptation and Profiling for Social Robotics in Rehabilitation [47]	2020	NAO	3	Cerebral palsy and brachial plexus palsy	7–9 years	Improve rehabilitation for children with cerebral palsy using NAO, adapting therapy sessions based on patient needs	System achieved high accuracy in posture detection (85%), improving motivation and simplifying clinician workload
“Evaluating the Child–Robot Interaction of the NAOTherapist Platform in Pediatric Rehabilitation [48]	2017	NAO	3	Motor disabilities	7–9 years	Evaluate the interaction between NAO and children during upper-limb rehabilitation, monitoring pose accuracy	Children demonstrated high engagement; system worked autonomously without human intervention
Adapting a General-Purpose Social Robot for Paediatric Rehabilitation through In Situ Design [38]	2018	NAO	9	Cerebral palsy	4–12 years	Adapt NAO for pediatric rehabilitation, reducing therapist intervention and improving patient compliance	Robot improved motivation and reduced session downtime; some technical issues reported (e.g., speech recognition)
Design, Development, and Evaluation of an Interactive Personalized Social Robot to Monitor and Coach Post-Stroke Rehabilitation Exercises [49]	2023	NAO	15	Stroke	55 years	Design a personalized social robot for stroke survivors, providing real-time feedback and monitoring exercises	System showed 81% accuracy in movement monitoring; participants reported increased confidence in performing exercises
Robot ZORA in Rehabilitation and Special Education for Children with Severe Physical Disabilities: A Pilot Study [39]	2017	ZORA	17	Sever physical disabilities	31 months–18 years	Explore the feasibility and usability of ZORA in rehabilitation and special education for children with disabilities	Improved motor, communication, and cognitive skills, though interest in the robot declined over time
A motor learning therapeutic intervention for a child with cerebral palsy through a social assistive robot [37]	2019	NAO	1	Dyskinetic cerebral palsy	8 years	Evaluate NAO’s role in improving motor learning in a child with cerebral palsy during walking exercises	Child improved walking performance, increasing steps without falling from 18 to 31 over 16 sessions
Hands-Off Therapist Robot Behavior Adaptation to User Personality for Post-Stroke Rehabilitation Therapy [42]	2007	NAO	11	Healthy participants	19–35 years	Design a robot that adapts feedback based on user personality (introvert/extrovert) during post-stroke rehabilitation	Robot adapted its behavior to user personality, leading to improved exercise performance. Extroverts preferred faster interactions
The Effect of Robot vs. Human Corrective Feedback on Children’s Intrinsic Motivation [43]	2019	NAO	10	-	4–8 years	Compare the motivational impact of corrective feedback from NAO vs. human therapists in a VR rehabilitation game	Children receiving feedback from NAO reported higher motivation compared to human feedback; however, differences were not significant
The Design of Lil’Flo, a Socially Assistive Robot for Upper Extremity Motor Assessment and Rehabilitation in the Community via Telepresence [44]	2021	Lil’Flo	13	Clinicians	-	Test Lil’Flo’s usability in upper extremity motor assessment and telepresence-based rehabilitation	Clinicians found Lil’Flo promising for remote rehabilitation, more interactive than traditional telepresence systems
Socially Assistive Robot for Stroke Rehabilitation: A Long-Term in-the-Wild Pilot Randomized Controlled Tria [45]	2024	Pepper	33	Post-stroke	58 years	Evaluate Pepper’s long-term role in providing feedback and monitoring in upper-limb post-stroke rehabilitation	Patients in the robot group showed significant improvements in clinical measures like FMA-UE and ARAT
Socially Assistive Robotics for Post-Stroke Rehabilitation [46]	2007	-	6	Post-stroke	-	Evaluate a non-contact SAR in motivating stroke patients to use their affected arm, improving adherence to exercises	Robot improved motivation and adherence to exercises, offering feedback without physical contact
Child and Parent Perceptions of Acceptability and Therapeutic Value of a Socially Assistive Robot Used During Pediatric Rehabilitation [40]	2019	NAO	5	Cerebral palsy	6–12 years	Assess NAO’s acceptability and therapeutic value during pediatric rehabilitation by parents and children	Children and parents found the robot acceptable and motivating. Some preferred human interaction
Potential Use of Social Assistive Robot Based Rehabilitation for Children with Cerebral Palsy [41]	2022	NAO	2	Cerebral palsy	5–14 years	Evaluate the use of NAO in improving participation and motivation in children with cerebral palsy during rehabilitation	Children showed increased participation and motivation during therapy sessions with NAO

## 4. Discussion

This review highlights the versatility of SARs across rehabilitation contexts. Their ability to motivate patients, guide exercises through imitation, and provide precise real-time feedback underscores their potential as powerful tools to enhance therapeutic adherence. The analyzed studies consistently demonstrate that SARs can reduce therapist workload, foster patient independence, and improve clinical metrics, making them integral to modern rehabilitation practices. The statistical distribution of studies across different pathologies (40% neurological disorders, 25% motor disabilities, 20% cardiovascular rehabilitation, and 15% other conditions) highlights the areas where SARs have been most widely applied. This distribution reflected the broad applicability of SARs in rehabilitation, with particular emphasis on neurological and motor disorders where the potential for therapeutic assistance was most pronounced. Additionally, 70% of the studies reported substantial improvements in rehabilitation outcomes, underscoring the general effectiveness of SARs in enhancing recovery. A further 20% of the studies showed moderate improvements, while 10% found no measurable effects, indicating that SARs are highly effective in certain contexts, but the overall efficacy can vary depending on the pathology and the specific rehabilitation goals.

The integration of SARs into rehabilitation settings has shown significant promise across various applications, from enhancing patient motivation to demonstrating exercises and providing real-time feedback. By classifying SARs into three main roles—Motivation, Imitation, and Feedback Providers—it becomes clear that robots like NAO, Pepper, ZORA, and others are versatile tools that can be tailored to meet the needs of both clinical and home-based rehabilitation environments. The distribution of these roles across studies (with eight studies focusing on motivation, four on imitation, and eight on feedback provision) highlights the different functions SARs fulfill in rehabilitation settings. SARs are not only effective in promoting engagement and adherence to therapy but also offer precise guidance and correction, helping patients achieve better outcomes with reduced reliance on constant human supervision. This functionality could be particularly beneficial in scenarios where patients are required to complete long-term rehabilitation regimens or exercises independently. With ongoing technological advancements, SARs hold great potential to revolutionize rehabilitation by offering more personalized and efficient therapeutic interventions. As technology continues to evolve, SARs could become central to the future of rehabilitation, offering tailored solutions that meet the unique needs of individual patients while maximizing the efficiency and effectiveness of rehabilitation therapies.

Despite these promising findings, a significant gap remains in the application of SARs for musculoskeletal disorders, particularly in orthopedic rehabilitation. The review revealed that a notable 70% of the studies focused on neurological and pediatric populations, while the application of SARs in musculoskeletal conditions remains underexplored. While SARs have demonstrated efficacy in neurological and pediatric rehabilitation, there is a lack of research on their use in musculoskeletal conditions (e.g., rotator cuff injuries, osteoarthritis) and post-surgical rehabilitation. Addressing this gap presents a substantial opportunity for future research and development.

Orthopedic rehabilitation, with its emphasis on restoring muscle strength, joint mobility, and motor control, presents unique challenges that SARs could effectively address. Robots capable of providing real-time feedback and personalized guidance could significantly enhance the quality and precision of rehabilitation exercises. Moreover, SARs could support patients in maintaining adherence to long-term rehabilitation programs, which is often a critical factor in orthopedic recovery. Considering the diversity of the reviewed studies, which focused on various pathologies and outcomes, applying SARs in musculoskeletal rehabilitation could lead to tailored solutions for specific patient needs. Future research should focus on developing and clinically validating SAR-based interventions for musculoskeletal rehabilitation, examining their effectiveness, cost-efficiency, and patient acceptance in orthopedic settings.

While SARs have shown substantial benefits in neurological and pediatric rehabilitation, their application in musculoskeletal and orthopedic rehabilitation remains underexplored [50,51,52]. Expanding the use of SARs in this area could offer significant clinical and economic benefits, improving patient outcomes and reducing the burden on healthcare providers. Addressing this research gap could pave the way for more comprehensive and effective rehabilitation strategies for musculoskeletal disorders.
